# Ameliorative Effects of Bredemolic Acid on Markers Associated with Renal Dysfunction in a Diet-Induced Prediabetic Rat Model

**DOI:** 10.1155/2020/2978340

**Published:** 2020-06-22

**Authors:** Akinjide Moses Akinnuga, Angezwa Siboto, Bongiwe Khumalo, Ntethelelo Hopewell Sibiya, Phikelelani Ngubane, Andile Khathi

**Affiliations:** ^1^Department of Physiology, School of Laboratory Medicine and Medical Sciences, College of Health Sciences, University of KwaZulu-Natal, Westville, Durban, South Africa; ^2^Department of Pharmacy and Pharmacology, Rhodes University, Grahamstown, South Africa

## Abstract

Recently, studies have shown that renal dysfunction is associated not only with overt diabetes but also with the preceding stage known as prediabetes. Diet and pharmacological interventions are the therapeutic approaches to managing prediabetes, but the compliance in combining the two interventions is low. Hence, the efficacy of pharmacological intervention is reduced without diet modification. In our previous study, we established that bredemolic acid (BA) ameliorated glucose homeostasis via increased GLUT 4 expression in the skeletal muscle of prediabetic rats in the absence of diet intervention. However, the effects of bredemolic acid on renal function in prediabetic condition are unknown. Therefore, this study was aimed at investigating the ameliorative effects of bredemolic acid on renal dysfunction in a diet-induced prediabetic rat model. Thirty-six Sprague-Dawley male rats (150–180 g) were divided into two groups: the nonprediabetic (*n* = 6) and prediabetic (*n* = 30) groups which were fed normal diet (ND) and high-fat high-carbohydrate (HFHC) diet, respectively, for 20 weeks. After the 20^th^ week, the prediabetic groups were subdivided into prediabetic control (PD) and 4 other prediabetic groups which were treated with either BA (80 mg/kg) or metformin (MET, 500 mg/kg) for further 12 weeks (21^st^ to 32^nd^). Plasma, urine, and kidney samples were collected for biochemical analysis. The untreated prediabetic (PD) rats presented increased fluid intake and urine output; increased creatinine, urea, and uric acid plasma concentrations; albuminuria; proteinuria; sodium retention; potassium loss; increased aldosterone and kidney injury molecule (KIM-1) concentration; and increased urinary podocin mRNA expression. However, BA administration attenuated the renal markers and oxidative stress and decreased the urinary podocin mRNA expression. In conclusion, BA administration, regardless of diet modification, attenuates renal dysfunction in an experimentally induced prediabetic state.

## 1. Introduction

More than 25% of type 1 and type 2 diabetes mellitus patients have been reported to develop renal dysfunction [1, 2]. However, the renal dysfunction does occur not only in overt diabetes but also in the early stages of impaired glucose metabolism [3, 4]. Renal dysfunction is defined by the appearance of abnormal kidney functional changes such as a reduced glomerular filtration rate (GFR), increased serum creatinine and urea, albuminuria, increased excretion of kidney injury molecule (KIM-1), and glomerular podocyte injury with urinary loss of podocin. Podocin is an exclusive integral membrane protein in the podocytes that directly interact with nephrin and CD2-associated protein [5]. Hence, urinary loss of podocin is an apparent indication of podocyte injury and renal dysfunction [6–8].

Moreover, literatures have shown that impaired glucose metabolism promotes renal dysfunction via activation of oxidative stress and renin-angiotensin-aldosterone system (RAAS) [9–11]. The activation of RAAS triggers the release of aldosterone which stimulates serum/glucocorticoid-regulated kinase 1 (SGK1) that regulate epithelial sodium channel (ENaC) and consequently lead to sodium retention and potassium loss in diabetic conditions [12–14]. Of note, literature evidence showed that about one-third of individuals with newly diagnosed diabetes mellitus have varying degrees of renal dysfunction [15]. This can only be attributed to the abnormal changes that occur during prediabetes. The prediabetic stage often precedes the onset of type 2 diabetes mellitus and is said to be caused by chronic consumption of high-caloric diets coupled with a sedentary lifestyle [15, 16]. Cross-sectional clinical studies have confirmed that prediabetes is associated with the onset of chronic kidney disease (CKD) [4, 17]. Therefore, screening of markers of renal function during the prediabetic state offers an early window of opportunity of preventing and managing CKD [15]. More importantly, diet modification and pharmacological intervention have been reported as the therapeutic approaches to managing prediabetes [18–20]. However, the compliance of combining the two interventions is low as patients adhere to pharmacological intervention without diet modification, and consequently, the efficacy of the pharmacological intervention is reduced [21, 22]. Hence, antidiabetic agents that can possibly ameliorate CKD regardless of diet intervention are considered necessary.

Studies in our laboratory have demonstrated that pentacyclic triterpenes, such as oleanolic acid, ursolic acid, and maslinic acid, are antidiabetic agents which attenuate renal dysfunction in streptozotocin-induced diabetes mellitus [23, 24]. Similarly, we have previously demonstrated that a maslinic acid isomer, bredemolic acid, is an antihyperglycaemic agent that regulated blood glucose concentration via increased expression of GLUT 4 in the gastrocnemius muscle of the prediabetic rat model without diet intervention [25]. However, the biological effects of bredemolic acid on renal dysfunction in the prediabetic state are unknown. Therefore, this study sought to investigate the effects of bredemolic acid on selected markers of renal function in a diet-induced prediabetic rat model, and we also treated the prediabetic rats with metformin, a common first-line drug in the therapy of type 2 diabetes and obesity [26].

## 2. Materials and Methods

### 2.1. Animals

Thirty-six (36) male Sprague-Dawley rats with body weight 150–180 g were used for this study as described in previous research [25]. The rats were obtained from the Biomedical Research Unit (BRU), University of KwaZulu-Natal (UKZN). The animals were kept and maintained in a standard animal facility under controlled environmental conditions at room temperature (22 ± 2°C), humidity (55 ± 5%), and 12 h day : 12 h night cycle. The animals consumed standard rat chow (Meadow Feeds, South Africa) and water *ad libitum* for 2 weeks to acclimatize before being exposed to the experimental diet (high-fat high-carbohydrate). The components of the high-fat high-carbohydrate (HFHC) diet are carbohydrate (55%%kcal/g), fats (30% kcal/g), and proteins (15% kcal/g) as described in the previous research [22]. All experimental procedures in this study were carried out in absolute compliance with the animal care guidelines and approved with ethical number (AREC/024/018D) by the Animal Research Ethics Committee (AREC) of the UKZN, Durban, South Africa.

### 2.2. Experimental Design

After the acclimatization, the animals were divided into 2 main groups: the nonprediabetic control group (*n* = 6) and the prediabetic group (*n* = 30). The nonprediabetic (NPD) control animals (negative control) were given standard rat chow (ND) and water *ad libitum* for 20 weeks while the prediabetic animals were given HFHC diet and drinking water supplemented with fructose (15%) for 20 weeks to induce prediabetes. At the 20^th^ week, prediabetes was confirmed via assessment of fasting blood glucose and oral glucose tolerance test (OGTT) as described by the American Diabetes Association and our previous study [25, 27]. Of notes, this study is a continuation of the previous study, and the data on body weight, food intake, fasting blood glucose, oral glucose tolerance test, fasting insulin concentration, and insulin resistance in the previous study are relevant for this present study.

### 2.3. Treatment of Animals

The treatment period lasted for 12 weeks (21^st^–32^nd^). The nonprediabetic control group (Group 1) fed on standard rat chow (ND) without treatment for 12 weeks while the prediabetic animals (*n* = 30) were further divided into the 5 groups (Group 2–Group 6, *n* = 6) and fed on HFHC or ND for 12 weeks as well. Group 2 served as the prediabetes control group (PD) and continuously fed on the HFHC diet without treatment for 12 weeks. The other 4 groups of the prediabetic animals continuously fed on HFHC diet or switched to ND and were treated with either oral administration of BA (80 mg/kg) or metformin (MET, 500 mg/kg) every third day for 12 weeks due to the three-day pharmacokinetic activity of pentacyclic triterpenes as previously described [28, 29]. The switch of diet from HFHC to ND is the dietary intervention while the continuous feeding on HFHC diet is the absence of dietary intervention. The ND+MET (Group 3) rats changed diet from HFHC to ND and received MET orally whereas the HFHC+MET (Group 4) rats were continuously fed on the HFHC diet and received MET orally. The ND+BA (Group 5) rats changed diet from HFHC to ND and received BA orally while HFHC+BA (Group 6) rats continuously fed on the HFHC diet and were treated with BA. After the 12 weeks of treatment, the animals were sacrificed*;* blood samples and the kidneys were collected from all the animals for biochemical analysis. The fluid intake and urine volumes were assessed in all the animals at the 20^th^ week and every 4 weeks (24^th^, 28^th^, and 32^nd^ week). The renal function parameters and other biochemical parameters were measured at the end of the experiment.

### 2.4. Determination of Fluid Intake and Urine Output

At the 20^th^ week and every 4 weeks thereafter, all the animals in each group were placed in different metabolic cages for 24 hours to measure fluid intake and urine output. The urine samples were measured and centrifuged at 13000 rpm for 5 minutes at 4°C, and the supernatants were stored at -80°C in a Bio Ultra freezer (Snijders Scientific, Tilburg, Holland) until ready for kidney function parameter analysis.

### 2.5. Blood Collection and Tissue Harvesting

All the animals were placed in a gas anaesthetic chamber (Biomedical Research Unit, UKZN, Durban, South Africa) and anaesthetised with 100 mg/kg of Isofor (Safeline Pharmaceuticals (Pty) Ltd., Roodeport, South Africa). In an unconscious state, blood samples were collected from all the animals via a cardiac puncture into different precooled EDTA containers. The blood samples were centrifuged (Eppendorf centrifuge 5403, Germany) 503 g for 15 minutes at 4°C to obtain plasma. Thereafter, the plasma samples were aspirated into plain sample bottles and stored in a Bio Ultra freezer (Snijders Scientific, Tilburg, Holland) at -80°C until ready for biochemical analysis. Also, the kidneys were removed, rinsed with cold normal saline solution, weighed on the weighing balance, snapped frozen in liquid nitrogen, and stored at -80°C in a Bio Ultra freezer for biochemical analysis of selected parameters.

### 2.6. Biochemical Analysis

The biochemical analysis of kidney function parameters (such as creatinine, urea, uric acid, albumin, and total protein) and electrolytes (Na^+^ and K^+^) was determined at the 32^nd^ week in the plasma and urine samples by using their respective assay kits (Elabscience Biotechnology Co., Ltd., Houston, TX, USA) as instructed by the manufacturer. However, the kidney injury molecule (KIM-1) and aldosterone plasma concentrations were determined from their specific ELISA kits as instructed by the manufacturer (Elabscience Biotechnology Co., Ltd., Houston, TX, USA) via the microplate reader, SPECTROstar Nano spectrophotometer (BMG LABTECH, Ortenburg, LGBW, Germany).

### 2.7. Determination of GFR

The GFR of all the animals were determined at the 32^nd^ week of the experiment from the estimation of creatinine in the plasma and urine (creatinine clearance) as follows:

(1) $$\begin{array}{c} \text{GFR}\left[\text{mL}/\min\right]=\frac{\text{Urine creatinine }\left(\text{mg}/\text{dL}\right)\times 24\text{hrs urine volume }\left(\text{mL}\right)}{\text{Plasma creatinine }\left(\text{mg}/\text{dL}\right)\times 60\min\times 24\text{hrs}}. \end{array}$$

### 2.8. Lipid Peroxidation and Antioxidant Status

The lipid peroxidation was assessed by determination of the concentration of malondialdehyde (MDA) in the kidney homogenized tissue according to the previously established protocol [24]. However, the antioxidant status of the kidney homogenate was assessed by determination of the concentration of superoxide dismutase (SOD), glutathione peroxidase (GPx), and total antioxidant capacity (TOAC) by using their specific ELISA kits according to the instruction of the manufacturer (Elabscience Biotechnology Co., Ltd., Houston, TX, USA).

### 2.9. Urine RNA Isolation

RNA was isolated from urine (4 mL) by using a ZR Urine RNA Isolation Kit™ (Zymo Research Corp., Irvine, USA) according to the manufacturer's protocol. The purity of the RNA was confirmed by the relative absorbance of ratio 260/280 nm via a Nanodrop 1000 spectrophotometer (Thermo Scientific, USA). Urine RNA (100 ng) was reverse transcribed to complementary DNA (cDNA) by using the iScript™ cDNA Synthesis Kit (Bio-Rad, California, USA) through incubation in a thermal cycler (SimpliAmp Thermal Cycler, Applied Biosystems, Life Technologies).

### 2.10. Urine Complementary DNA (cDNA) Synthesis

For cDNA synthesis, urine RNA (2 *μ*L) was mixed with 5x iScript reaction (4 *μ*L), iScript reverse transcriptase enzyme (1 *μ*L) (Bio-Rad, USA), and nuclease-free water to a final volume of 20 *μ*L. The mixture was incubated in the thermal cycler (SimpliAmp Thermal Cycler, Applied Biosystems, Life Technologies) at 25°C for 5 minutes, 42°C for 30 minutes, and finally at 85°C for 5 minutes. Thereafter, the synthesized cDNA was stored at -80°C until use for real-time PCR (polymerase chain reaction).

### 2.11. Real-Time PCR

The urinary mRNA level of podocin was quantified by real-time PCR LightCycler (Roche LightCycler 96, USA). cDNA template (2 *μ*L), SYBR Green PCR master mix (5 *μ*L) (Bio-Rad, USA), podocin forward primer (1 *μ*L), podocin reverse primer (1 *μ*L), and nuclease-free water were mixed to a final volume of 10 *μ*L. Thereafter, the sample mixtures were cycled 40 times at 95°C for 10 seconds, 60°C for 20 seconds, and 72°C for 20 seconds in the LightCycler (Roche LightCycler 96, USA). All the samples were run in duplicate, and *β*-actin mRNA levels were used as a housekeeping gene to normalize the podocin mRNA level. The sequences of the used oligonucleotide primers (Metabion International AG, Planegg, Germany) were as follows: *podocin forward 5*′*-TGG AAG CTG AGG CAC AAA GA-3*′ and *podocin reverse 5*′*-AGA ATC TCA GCC GCC ATC CT-3*′.

### 2.12. Statistical Analysis

The data were normally distributed and presented as the mean ± SEM. Multiple intergroup differences were analysed by one-way ANOVA with the Bonferroni test as a post hoc test through GraphPad Prism 7 software. The results were considered statistically significant at *p* < 0.05.

## 3. Results and Discussion

### 3.1. Effects of BA Administration with or without Diet Intervention on Plasma and Urinary Albumin and Total Protein

**Figure 1:**
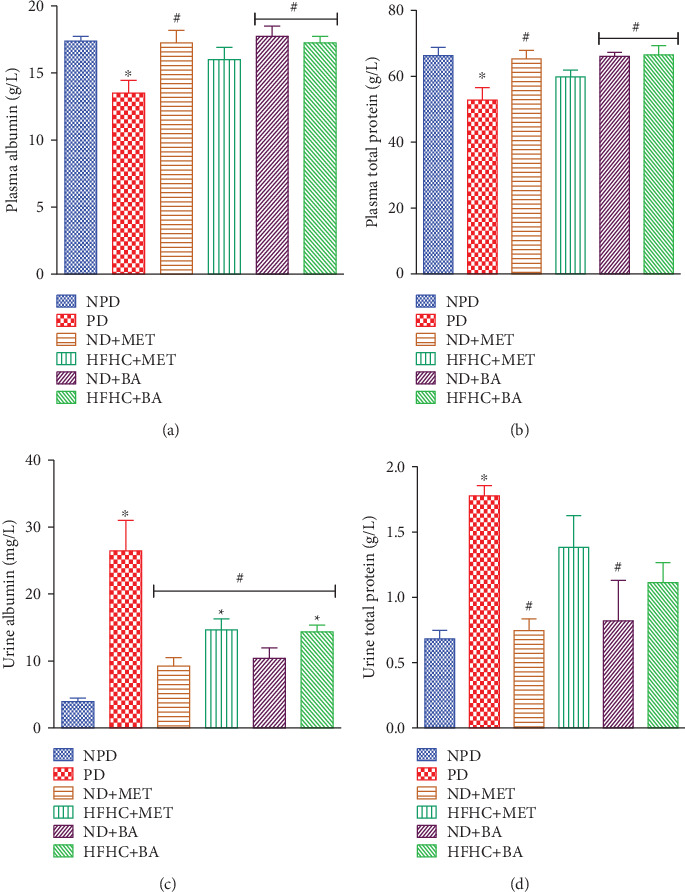
Effects of BA on plasma albumin (a), plasma total protein (b), urine albumin (c), and urine total protein (d) in prediabetic rats in the presence or absence of dietary intervention. ^∗^*p* < 0.001 (vs. NPD), ^#^*p* < 0.05 (vs. PD).

High-fat or high-fructose diet has been associated with impaired glucose metabolism and insulin resistance which in turn leads to metabolic disturbances with complications that result in renal dysfunction such as decreased plasma concentration of albumin and total protein, albuminuria, proteinuria, and diffuse thickening of the glomerular capillary basement membrane [30–33]. In this study, a significant decrease in plasma concentrations of albumin (Figure 1(a)) and total protein (Figure 1(b)) was observed in the prediabetic control rats when compared to nonprediabetic control rats. In addition, albuminuria (Figure 1(c)) and proteinuria (Figure 1(d)) which are apparent indicators of renal damage were observed in the prediabetic control rats compared to nonprediabetic rats. These observations may be attributed to the impaired filtration barrier which has been reported in prediabetic condition in other studies [30, 34]. Therefore, we suggest that the abnormal glucose homeostasis and insulin resistance that are associated with prediabetes due to chronic consumption of high-caloric diet might have resulted into the impaired filtration barrier with consequent loss of plasma albumin and protein, thus resulting into significant albuminuria and proteinuria [3, 35]. However, the administration of BA in the presence or absence of diet intervention as well as metformin administration with diet intervention attenuated albuminuria and proteinuria in the BA- and metformin-treated prediabetic rats, and this in turn contributed to the improved plasma concentrations of albumin (Figure 1(a)) and total protein (Figure 1(b)). We therefore suggest that BA attenuated these renal dysfunction markers by its antihyperglycaemic property and the improved insulin sensitivity which we have earlier reported in our study [25].

### 3.2. Effects of BA Administration with or without Diet Intervention on Plasma KIM-1

**Figure 2:**
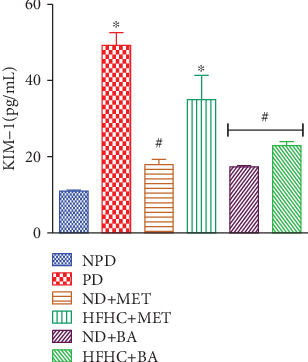
Effects of BA on plasma kidney injury molecule (KIM-1) concentrations in prediabetic rats in the presence or absence of dietary intervention.

Apart from albuminuria or proteinuria, another indicator of renal damage is the KIM-1, which is an expressed biomarker on the apical membrane of proximal tubular cells [36]. The observed significant increase in the plasma concentration of KIM-1 in the prediabetic control rats compared to nonprediabetic control rats in this study (Figure 2) was also an indication of decline in renal function, and this observation on KIM-1 correlated with other studies in insulin-resistant states [37, 38]. However, the KIM-1 plasma concentration of BA-treated prediabetic rats with or without dietary intervention as well as metformin-treated prediabetic rats with diet intervention was significantly decreased in comparison to the prediabetic control rats.

### 3.3. Effects of BA Administration with or without Diet Intervention on Plasma and Urinary Uric Acid and Urea

**Figure 3:**
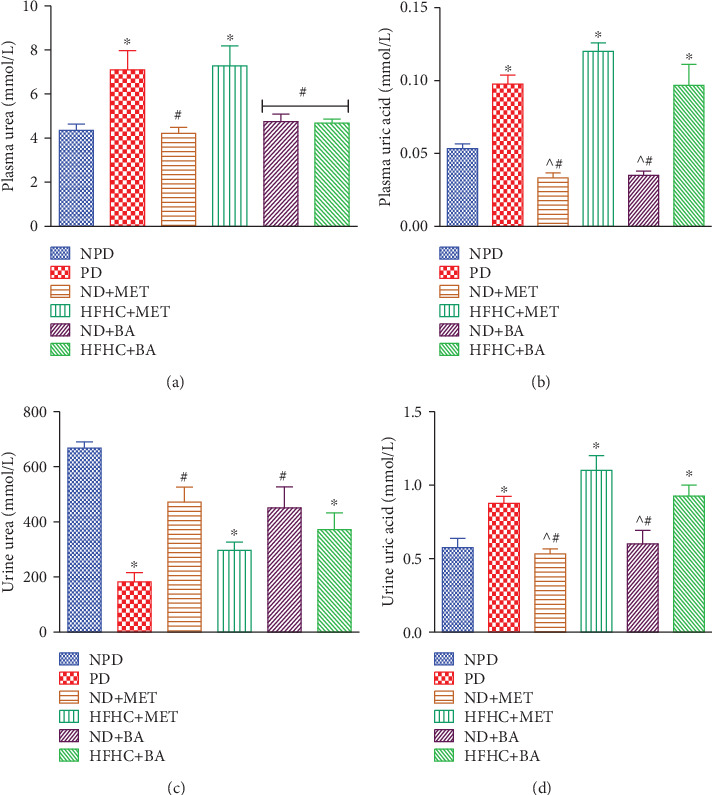
Effects of BA on plasma urea (a), plasma uric acid (b), urine urea (c) and urine uric acid (d) in prediabetic rats in the presence or absence of dietary intervention. ^∗^*p* < 0.001 (vs. NPD), ^#^*p* < 0.001 (vs. PD), and ^^^*p* < 0.001 (vs. HFHC+MET).

In this study, the plasma concentrations of urea and uric acid (Figure 3(a) and Figure 3(b), respectively) significantly increased in the prediabetic control rats when compared to the nonprediabetic control rats. The alterations in the plasma or urinary concentrations of urea may be suggested to be due to impaired excretory or regulatory function of the kidney in maintaining constant homeostasis in the prediabetic or diabetic state [39]. Moreover, decreased urinary concentration of urea (Figure 3(c)) in the prediabetic control rats in comparison to the nonprediabetic control rats was observed in this study. This observation was in accordance with the results of previous studies [24, 40]. Administration of BA in the absence or presence of dietary intervention as well as metformin in the presence of dietary intervention significantly decreased the plasma and increased the urinary concentrations of urea.

Of note, high fructose diet has been reported to result in ATP depletion due to utilization of two molecules of ATP for each fructose molecule metabolized [41, 42]. Therefore, the resultant ADP is further degraded to AMP. In the insulin-resistant state (prediabetes), xanthine dehydrogenase enzyme is activated and triggered the conversion of the AMP to uric acid, hence resulting into the observed hyperuricaemia and elevated uric acid excretion in this study [43, 44]. Therefore, we suggest that the significant increase in uric acid levels in the plasma may be due to the chronic consumption of fructose diet which triggered insulin resistance and further leads to the observed hyperuricaemia and significant urinary excretion of uric acid in prediabetic control rats (Figure 3(d)). However, we hypothesized that the administration of BA and metformin in the presence of dietary intervention significantly ameliorated the hyperuricaemia probably due to the improved insulin sensitivity in the BA- and metformin-treated prediabetic rats.

### 3.4. Effects of BA Administration with or without Diet Intervention on Lipid Peroxidation and Antioxidant Status in the Kidney

The observed increase in the lipid peroxidation (MDA) and decrease in the concentration of antioxidant enzymes (SOD, GPx, and TOAC) in the prediabetic control rats in comparison to the nonprediabetic control rats are apparent indicators of oxidative stress (Table 1). Increased glucose influx into the cells (due to consumption of high-caloric diet) which results into increased glucose catabolism through the Krebs cycle and production of electron donors (NADH and FADH2) at quantities that overwhelm the capacity of oxidative phosphorylation electron transport chain triggers oxidative stress under hyperglycaemic conditions [45]. This process occurs in microvascular endothelial cells such as the glomerular endothelial cells which are unable to decrease glucose influx during a hyperglycaemic state [46]. The glomerular endothelium plays a significant role in the pathogenesis of diabetic nephropathy directly and through its interaction with podocytes [45]. Therefore, we suggest that another mechanism for the antioxidant effect of BA may probably be due to the decreased postmeal glucose in BA-treated prediabetic animals.

### 3.5. Effects of BA Administration with or without Diet Intervention on Plasma, Urine Creatinine, and GFR

**Figure 4:**
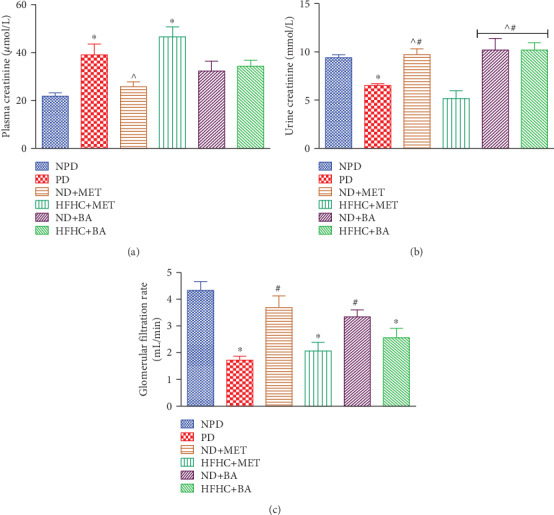
Effects of BA on plasma creatinine (a), urine creatinine (b), and GFR (c) in prediabetic rats in the presence or absence of dietary intervention. ^∗^*p* < 0.001 (vs. NPD), ^#^*p* < 0.001 (vs. PD), and ^^^*p* < 0.01 (vs. HFHC+MET).

The plasma concentrations of creatinine significantly increased (Figure 4(a)) while the urinary concentration of the same parameter (Figure 4(b)) in the prediabetic control rats was significantly decreased in comparison to the nonprediabetic control rats. These observations were correlated with the results of other studies [24, 40]. The impaired creatinine clearance altered the plasma and urine creatinine concentrations and further contributed to the decreased GFR in the prediabetic control rats (Figure 4(c)) [2]. Studies have shown that insulin resistance triggers oxidative stress in renal tissues [47, 48]. Therefore, we suggest that the impaired creatinine clearance which resulted into the decreased GFR may be due to insulin resistance which further triggered oxidative stress as reported in other studies [49, 50]. However, the administration of BA in the absence or presence of diet intervention and metformin administration in the presence of diet intervention significantly increased the urine creatinine by comparison to the prediabetic control rats. Also, the GFR of BA and metformin-treated prediabetic rats with diet intervention significantly increased by comparison to the PD control rats (Figure 4(c)). Therefore, we suggest that the improved creatinine clearance in BA-treated prediabetic rats is due to the antioxidant activity of the pentacyclic triterpene.

### 3.6. Effects of BA Administration with or without Diet Intervention on Plasma Aldosterone

**Figure 5:**
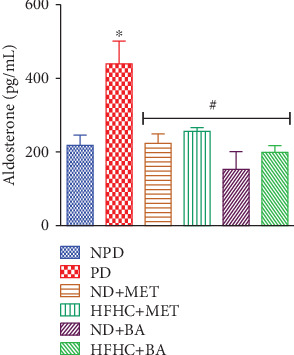
Effects of BA on plasma aldosterone concentrations in prediabetic rats in the presence or absence of dietary intervention. ^∗^*p* < 0.001 (vs. NPD), ^#^*p* < 0.001 (vs. PD).

A high-fat diet has been reported to activate the renin-angiotensin-aldosterone system (RAAS) in insulin-resistant states [14, 20, 30]. Also, literatures have shown that due to hyperinsulinaemia in insulin-resistant states, aldosterone production increases, and this in turn activates the aldosterone-induced SGK1 signaling pathway [13, 51]. In correlation with other studies [40, 52], significantly elevated plasma concentration of aldosterone was also observed in the prediabetic control rats when compared to the nonprediabetic control rats (Figure 5). Therefore, we suggest that the consumption of the high-fat diet contributed to the elevated aldosterone concentration through the activation of RAAS in the prediabetic control rats. In this study, the administration of BA and metformin in the absence or presence of diet intervention significantly decreased the plasma aldosterone concentration in the BA- and metformin-treated prediabetic rats. Therefore, we suggest that the administration of BA probably improved insulin sensitivity which in turn reduced the activation of RAAS and consequently leads to the significantly decreased plasma aldosterone concentration in BA-treated prediabetic rats even in the absence of diet intervention.

### 3.7. Effect of BA Administration with or without Diet Intervention on Plasma and Urinary Sodium and Potassium, Fluid Intake, and Urine Output

Due to the aforementioned RAAS activation and elevated plasma concentration of aldosterone in insulin-resistant states, the fluid intake, urine output, sodium reabsorption, and potassium loss significantly increased in the prediabetic control rats in this study. Literature has shown that the activation of RAAS subsequently activates the serum/glucocorticoid-regulated kinase 1 (SGK1) which further triggers the stimulation of the epithelial sodium channel (ENaC) to cause sodium retention, hypokalemia, and increased fluid intake [13, 51]. In this study, the fluid intake and urine output of the prediabetic control rats were significantly increased in comparison to the nonprediabetic control rats throughout the treatment period (Table 2). However, in the presence or absence of dietary intervention with BA administration as well as metformin administration with diet intervention, the fluid intake and urine output significantly decreased when compared to the prediabetic control rats, especially at the 12^th^ week period of treatment (*p* < 0.05).

**Figure 6:**
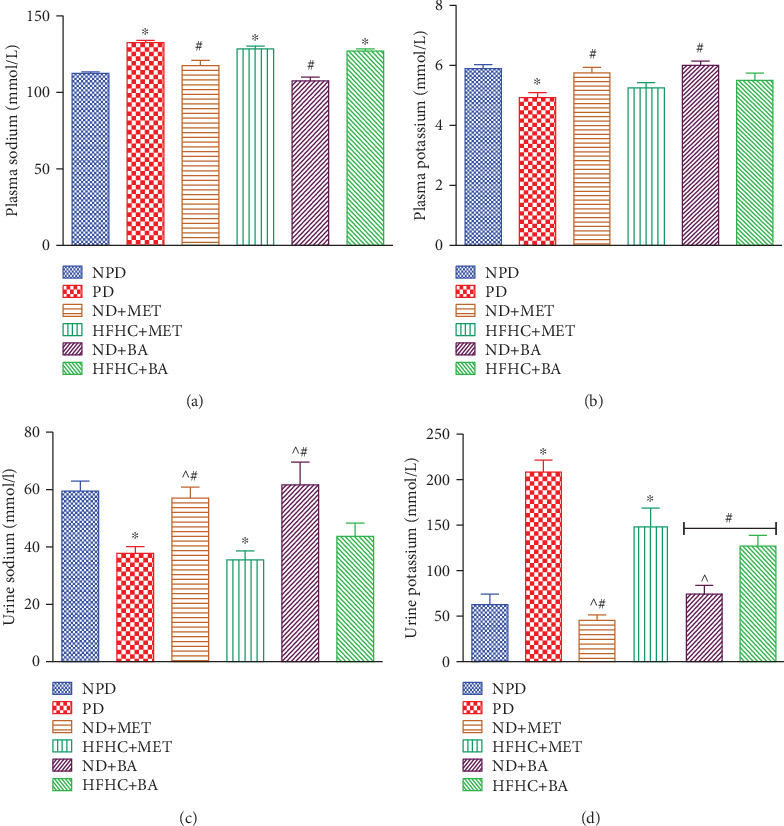
Effects of BA on plasma sodium (a), plasma potassium (b), urine sodium (c), and urine potassium (d) in prediabetic rats in the presence or absence of dietary intervention. ^∗^*p* < 0.001 (vs. NPD), ^#^*p* < 0.05 (vs. PD), and ^^^*p* < 0.001 (vs. HFHC+MET).

Moreover, the administration of BA or metformin with diet intervention significantly decreased the plasma sodium concentration (Figure 6(a)) and increased the plasma potassium concentration (Figure 6(b)) when compared to the prediabetic control rats (*p* < 0.05). On the other hand, the BA- or metformin-treated prediabetic rats with diet intervention had significantly increased urinary sodium (Figure 6(c)) and decreased urinary potassium (Figure 6(d)) by comparison to the prediabetic control rats. Apart from RAAS, other mechanisms that can possibly be associated with the increased fluid intake, urine output, and electrolyte imbalance in the prediabetic control rats are hyperglycaemia and glycosuria. Therefore, we suggest that the amelioration of fluid intake, urine output, and the electrolytes by administration of BA may be attributed to the improved hyperglycaemia and glycosuria in the BA-treated prediabetic rats as reported in the previous study [25].

### 3.8. Effect of BA Administration with or without Diet Intervention on Urinary Podocin mRNA Expression

**Figure 7:**
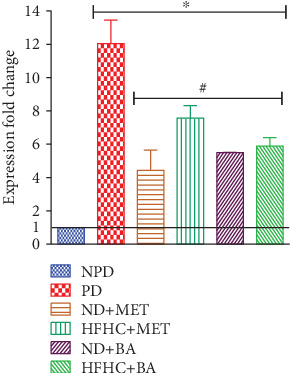
Effects of BA on urinary podocin mRNA expression in prediabetic rats in the presence or absence of dietary intervention. ^∗^*p* < 0.001 (vs. NPD), ^#^*p* < 0.001 (vs. PD).

Literature evidences revealed that elevated aldosterone concentration induced proteinuria and glomerular podocyte injury with decreased gene expression of podocin in the kidney tissues and increased gene expression of podocin mRNA in the urine [53, 54]. Also, it has been established that podocytes express mineralocorticoid receptors (MR); hence, podocytes are targeted cells for aldosterone hormone [53, 55]. Therefore, when aldosterone concentration is increased, oxidative stress is induced in the podocytes, and this subsequently promotes podocyte injury by increased reactive oxygen species (ROS) production in the mitochondria [56]. In addition, it has been demonstrated that podocytes are insulin-responsive cells that similarly respond to insulin in the same manner as the skeletal muscle [57]. This showed that podocyte survival is modulated by insulin signaling [57]. Similarly, in this study, the aforementioned increase in urinary podocin mRNA expression was observed in prediabetic control rats, and this correlated with other similar studies [8, 58]. The podocin mRNA expression in the urine of prediabetic control rats was significantly increased by 12.04-fold when compared to the nonprediabetic control rats (Figure 7). The podocin mRNA expressions in the urine of BA and metformin-treated prediabetic rats in the presence or absence of diet intervention were significantly decreased when compared to the prediabetic control rats.

However, we suggest that the administration of BA probably improved insulin sensitivity and ameliorated the insulin signaling in podocytes, and this further contributed to the observed decreased gene expression of urinary podocin mRNA in BA-treated prediabetic rats in this study. Moreover, pentacyclic triterpenes have been reported to selectively inhibit 11*β*-hydroxysteroid dehydrogenase type I enzyme, an enzyme that converts inactive cortisone into active cortisol, thus preventing activation of mineralocorticoid receptors in aldosterone tissue such as the kidney [59, 60]. Therefore, we hypothesized that the same enzymatic inhibition may probably prevent aldosterone biological actions on podocyte mineralocorticoid receptors and this subsequently led to reduced podocyte injury which in turn contributed to the decreased urinary gene expression of podocin mRNA in the BA-treated prediabetic rats with or without diet modification.

## 4. Conclusion

Administration of BA with or without diet modification has been shown in this study to attenuate renal dysfunction markers and urinary expression of podocin mRNA in the prediabetic state. These biological actions of BA may be due to the earlier reported combination of the improved insulin sensitivity, antihyperglycaemic and antioxidant properties of the pentacyclic triterpene (BA) [61, 62]. Pentacyclic triterpenes have been reported as nontoxic antioxidants that have low pharmacokinetic activity of three days without side effects [28, 29]. Therefore, we suggest that the ameliorative effects of BA on renal function markers compared to metformin in this study may be attributed to the low pharmacokinetic feature of BA even in the absence of dietary intervention. However, this is a preliminary study, more structural and molecular findings are still needed to clarify the mechanisms by which BA ameliorates renal function.

## Figures and Tables

**Table 1:** The effects of BA on lipid peroxidation and antioxidant status in prediabetic rats in the presence or absence of dietary intervention. Values are presented as the mean ± SEM (*n* = 6).

Groups	NPD	PD	ND+MET	HFHC+MET	ND+BA	HFHC+BA
Parameters						
MDA (nmol/g protein)	5.10 ± 0.13	7.72 ± 0.41^∗∗∗^	5.69 ± 0.19^##^	6.75 ± 0.40^∗∗^	5.07 ± 0.08^###^	5.63 ± 0.25^###^
SOD (ng/mL)	8.66 ± 0.27	3.14 ± 0.38^∗∗∗^	9.92 ± 0.52^###^	6.62 ± 0.12^###^	11.45 ± 0.63^∗^^###^	8.08 ± 0.81^###^
GPx (pg/mL)	1793.00 ± 42.38	849.27 ± 24.69^∗∗∗^	1820.11 ± 25.88^###^	1274.50 ± 36.14^∗∗∗^	1914.21 ± 37.18^###^	1698.61 ± 33.17^###^
TOAC (U/mL)	44.40 ± 2.57	14.80 ± 1.03^∗∗∗^	31.45 ± 1.02^∗^^###^	22.14 ± 3.03^∗∗∗^	41.31 ± 1.65^###^	24.17 ± 3.10^∗∗∗^^#^

^∗^ *p* < 0.05, ^∗∗^*p* < 0.01, and ^∗∗∗^*p* < 0.001 (vs. NPD); ^#^*p* < 0.05, ^##^*p* < 0.01, and ^###^*p* < 0.001 (vs. PD).

**Table 2:** Effects of BA on fluid intake and urine output in prediabetic rats in the presence or absence of dietary intervention. Values are presented as the mean ± SEM (*n* = 6).

Parameters	Groups					
	NPD	PD	ND+MET	HFHC+MET	ND+BA	HFHC+BA
*Fluid intake (mL)*						
0 week	21.50 ± 0.96	54.50 ± 4.54^∗^	59.00 ± 3.63^∗^	51.00 ± 4.73^∗^	66.17 ± 6.43^∗^	52.83 ± 5.10^∗^
4 weeks	23.17 ± 1.76	34.50 ± 4.07^∗^	22.67 ± 2.16^#^^	39.83 ± 7.10^∗^	25.00 ± 3.37^^^	30.33 ± 3.33
8 weeks	20.83 ± 2.39	35.00 ± 2.89^∗^	23.33 ± 3.33	32.5 ± 2.81	25.50 ± 2.93	33.33 ± 2.47
12 weeks	19.50 ± 1.38	34.17 ± 2.01^∗^	20.83 ± 1.54^#^	30.00 ± 2.24	22.50 ± 2.14^#^	22.17 ± 2.32^#^
*Urine output (mL)*						
0 week	8.67 ± 0.67	31.33 ± 3.82^∗^	33.17 ± 3.21^∗^	30.33 ± 2.60^∗^	39.00 ± 3.00^∗^	34.33 ± 3.77^∗^
4 weeks	9.00 ± 0.86	26.67 ± 3.41^∗^	17.00 ± 2.46^#^^	29.67 ± 2.89^∗^	17.67 ± 2.39^#^^	18.33 ± 2.45^∗^^^^
8 weeks	11.00 ± 0.45	23.33 ± 4.28^∗^	15.67 ± 1.75	20.83 ± 2.34^∗^	14.00 ± 2.00^#^	18.00 ± 2.00
12 weeks	11.00 ± 1.44	26.33 ± 2.03^∗^	16.67 ± 1.12^#^	22.17 ± 2.23^∗^	16.50 ± 1.67^#^	16.00 ± 2.19^#^

^∗^ *p* < 0.001 (vs. NPD), ^#^*p* < 0.05 (vs. PD), and ^^^*p* < 0.05 (vs. HFHC+MET).

## Data Availability

The data used to support our findings in this study are available upon request from the corresponding author. However, the data on body weight, food intake, fasting blood glucose, and oral glucose tolerance test have been reported in our previous study.
